# Preparation and Chemical and Physical Characteristics of an Edible Film Based on Native Potato Starch and Nopal Mucilage

**DOI:** 10.3390/polym13213719

**Published:** 2021-10-28

**Authors:** David Choque-Quispe, Sandro Froehner, Carlos A. Ligarda-Samanez, Betsy S. Ramos-Pacheco, Henry Palomino-Rincón, Yudith Choque-Quispe, Aydeé M. Solano-Reynoso, Fredy Taipe-Pardo, Lourdes Magaly Zamalloa-Puma, Miriam Calla-Florez, Miriam E. Obregón-Yupanqui, Miluska M. Zamalloa-Puma, Antonieta Mojo-Quisani

**Affiliations:** 1 Water Analysis and Control Research Laboratory, Universidad Nacional José María Arguedas, Andahuaylas 03701, Peru; bsramos@unajma.edu.pe; 2 Department of Environmental Engineering, Universidade Federal do Parana (UFPR), Curitiba 80060-000, Brazil; froehner@ufpr.br; 3 Food Nanotechnology Research Laboratory, Universidad Nacional José María Arguedas, Andahuaylas 03701, Peru; caligarda@unajma.edu.pe; 4 Agroindustrial Engineering, Universidad Nacional José María Arguedas, Andahuaylas 03701, Peru; hpalomino@unajma.edu.pe (H.P.-R.); ftaipe@unajma.edu.pe (F.T.-P.); 5 Environmental Engineering, Universidad Nacional José María Arguedas, Andahuaylas 03701, Peru; yuditchoque@gmail.com; 6 Department of Environmental Engineering, Universidad Tecnológica de los Andes, Apurímac 03001, Peru; ayma_21@hotmail.com; 7 Faculty of Engineering, Universidad Continental, Cusco 12000, Peru; magalyzamalloa@gmail.com (L.M.Z.-P.); ZMILUSKA@HOTMAIL.COM (M.M.Z.-P.); 8 Agroindustrial Engineering, Universidad Nacional de San Antonio Abad del Cusco, Cusco 08000, Peru; miriam.calla@unsaac.edu.pe (M.C.-F.); antonieta.mojo@unsaac.edu.pe (A.M.-Q.); 9 Agroindustrial Research Laboratory, Universidad Nacional José María Arguedas, Andahuaylas 03701, Peru; mirianobregon95@gmail.com

**Keywords:** solubility, water activity, nopal mucilage, native potato starch, Allcca sipas

## Abstract

Edible films prepared from biological materials are being massively used. This study aimed to prepare edible films from native potato starch of the Allcca sipas variety (*Solanum tuberosum* subsp. Andigena), nopal mucilage (*Opuntia ficus* indica), and glycerol. Twelve edible films were prepared with starch, mucilage, and glycerin in different proportions by thermosynthesis. It was observed that mucilage and glycerol have a significant direct effect on film solubility and an inverse effect on a_w_, while the effect of starch is the opposite. The a_w_ ranged from 0.562 to 0.639. The FTIR analysis showed the interaction of the components in the films being considerably influenced by the addition of mucilage. The TGA/DTA analysis reported low thermal stability in the films, retaining water around 100 °C, and showing a tendency to lose weight when the content of starch is high, while the opposite occurred with the addition of mucilage; it was also observed that around 310 °C, the maximum weight loss was observed between 53.6 and 86.1%. SEM images showed uniform films without cracks. The results are promising and show the possibility of preparing edible films from native potato starch and mucilage.

## 1. Introduction

In the post-harvest, the conservation of fruits and vegetables are made by edible films, which have become extensively used. Edible films are made from plant and animal materials, allowing to take advantage of its constituents, such as proteins, polysaccharides, lipids, and their mixtures [1]. The environmental friendly aspect of such films has given them an advantage in comparison the films derived from petroleum [2]. These films allow the extension of the shelf life of food, giving it the appearance of freshness, with good shine, and color, improving its commercial value through the control of gas transfer and growth of microorganisms [3], that in most cases accelerate their maturity and senescence [1,4,5].

Water activity is one of the important parameters in food preservation, especially in foods with high moisture and nutrient content, which can be attacked by microorganisms, and deteriorate their quality. Coatings have the ability to minimize or eliminate this fact, and this will depend on their composition and formulation; therefore, values lower than 0.65 are adequate to avoid the development of undesirable microorganisms [6].

On the other hand, the response of edible films to changes in temperature to which they may be subjected allows one to evaluate the availability of their constituents, mainly water and volatile compounds, which define the qualities of films. This behavior can be described through thermal analysis; however, the interaction of the constituents depends on the functional groups presented, such groups can be evidenced through an infrared study (IR) and the visualization of the microtopography of the film using scanning electron microscopy (SEM) [7,8,9].

A nopal (*Opuntia ficus* indica) is a cactus that grows in soils and demands low amounts of nutrients and humidity. This cactus has a mucilage with good rheological properties [10], and is made up of sugars, such as arabinose, rhamnose, galactose, xylose, pectins, and uronic acids [11,12], with disposition of functional hydroxyls and carbonyl groups, giving it a high ability to bind to polar macromolecules, in other words, creating qualities to form polymeric films [4,8,13]. Many attempts have been made to improve the physical and chemical properties of mucilage based on films, by adding carrageenans [14], solvents [15], microencapsulation in gallic acid [14], corn starch [10], pectin [16], polyvinyl alcohol, chitosan, sorbitol, glycerol [13].

The native potato (*Solanum tuberosum* subsp. Andigena) grows above 3500 m of altitude and is consumed by local people. This tuber has a high content of dry matter [17] of which starch is found in a higher percentage, with an oval and spherical shape, and a granule size greater than starches from other sources [18]. The native potato starch is constituted by amylose and amylopectin, the latter of which has a branched chain, which confers a greater number of hydroxyl groups and phosphate ester, making it responsible for having a better mechanical behavior [19] especially when linking with other molecules for the preparation of films [20]. Similarly, its low gelatinization temperature and the rheological behavior of pastes and gels allow for film conformation treatments at low temperatures [19,21,22].

The purpose of this work was to prepare an edible film formulated with native potato starch of the Allcca sipas variety, nopal mucilage, and glycerol as a plasticizer, and determine the physical and chemical characterization in order to apply as a coater for fruit and vegetable protection and conservation.

## 2. Materials and Methods

### 2.1. Vegetal Material

The native potato of the Allcca sipas variety (*Solanum tuberosum* subsp. Andigena) was collected in its commercial maturity state from the cultivation fields of the Chulcuisa town center in May 2018, located at 13°41′01″ S, 73°14′20″ W, and 3880 m of altitude, of the Andahuaylas Province, Peru. The wild cladodes of nopal (*Opuntia ficus* indica) were collected in Possoccoy area in June 2018, located at 13°35′26.4″ S, 73°27′0.8″ W, 2500 m altitude from the Talavera district, Andahuaylas, Peru.

### 2.2. Nopal Mucilage and Starch Extraction

The starch was obtained by hydroextraction and dried at 45 ± 2 °C for 14 h, then it was ground in a planetary ball mill, Resch PM10, at 150 rpm for 3 min and sieved to 250 µm. The mucilage was obtained by chopping the nopal cladodes, immersing them in water with a 1:1 ratio. Then, 24 h later the obtained liquid was treated with ethanol at 96°, in a 3:1 ratio, to extract the precipitated mucilage which was dried at 70 °C for 24 h. It was ground in a planetary ball mill, Resch PM10, at 150 rpm for 3 min and sieved to 250 µm, obtaining a fine powder.

### 2.3. Formulation of Edible Films

Films were formulated with native potato starch, powdered mucilage, and glycerol (G) (99.5%, Scharlau, Barcelona, Spain). A 2% starch solution (S) and 0.5% mucilage (M) were mixed, gelled at 70 °C with continuous stirring for each case. Subsequently, it was allowed to cool down to 40 °C and mixing was carried out according to the formulations in Table 1 in the order S-G-M. Stirring was constant at 40 rpm, maintaining the temperature for 5 min to guarantee the complete mix. A total of 50 mL of the mixture was charged in silicone molds, and placed in a forced convection dryer, Binder model FED, Tuttlingen, Germany, at the temperature of each formulation for 24 h.

### 2.4. Determination of Solubility and Resistance to Solvents

A film sample was taken in a ratio of 1 film: 100 solvent (g/mL), solvents were prepared with hydrochloric acid (pH 4.7), potassium hydroxide (pH 8.7), and ultrapure water; it was allowed to stand for 24 h, and the disintegrated fraction was passed through filter paper and dried at 105 °C for 24 h. Solubility was expressed in terms of the percentage of dry disintegrated material [1].

For solvent resistance, 0.5 cm × 1.0 cm films were cut and introduced in 20 mL of a solvent and left for 24 h at 20 °C. The solvent media was 0.01 M HCl, 0.01 M NaOH, 0.01 M CH_3_COOH, ethanol at 96°, petroleum ether, and deionized water. It was qualified as highly soluble (HS) when no film particles were observed in the solvent medium; moderately soluble (MS) when small particles of the film without disintegration were evident; low solubility (LS) when the films hardly disintegrated; and not soluble (NS) when the film remained intact in the solvent medium.

### 2.5. Determination of Water Activity (a_w_)

The samples were previously taken to a desiccator with silica gel for 48 h, a_w_ was determined through the AquaLab 4TEV equipment (Decagon Devices Inc., Washington, DC, USA).

### 2.6. FTIR Analysis

Pressed tablets were prepared, with 0.1% of the film in KBr (IR Grade, Darmstadt, Germany), and they were brought to the transmission module of the FTIR spectrometer (Fourier-transform infrared spectroscopy), Thermo Fisher (Waltham, MA, USA), Nicolet is 50 model, in a range of 4000 to 400 cm^−1^ with a resolution of 4 cm^−1^.

### 2.7. Thermal Analysis

The thermal stability of the films was determined with a TGA/DTA (TGA, thermogravimetric analysis, DTA, differential thermal analysis) analyzer model STA PT 1600-LINSEIS, Selb, Germany, in air atmosphere with a heating rate of 10 °C/min, temperature range of 18 to 630 °C, and alumina crucibles (Al_2_O_3_) were used as sample carriers.

### 2.8. SEM Analysis

A Quanta 200 model scanning electron microscope (SEM, Thermo Fisher, Waltham, MA, USA) was used. The films were fixed on an adhesive tape and taken to the vacuum chamber of the equipment, and the surface of the biopolymer was recognized through the equipment’s software.

### 2.9. Statistical Analysis

The extreme vertices mixture design was used and the data were collected in triplicate. An analysis of variance (ANOVA) was applied, as well as the Tukey’s mean test at 5% significance. Statistica 8.0 software (Statsoft Inc., Tulsa, OK, USA) was used for statistical analysis.

## 3. Results and Discussion

### 3.1. Solubility and Resistance to Solvents

The films subjected to the acid, basic, and neutral media did not report a significant difference in terms of solubility for each formulation in almost all cases (*p*-value > 0.05), however, this increased slightly at a higher treatment temperature. In Table 2, it is observed that the solubility is in the range of 19.77 to 54.08%. Dick et al. [1] reported solubility of up to 84.5% for films made with chia seed glycerol mucilage, and González et al. [16] reported 91.04% for nopal mucilage films, although the solubility behavior will depend on the use that is given to the edible film [3,23].

Films with formulation F3 reported higher solubility (Table 2), while formulation F2 and F4 showed lower values; that is, F3 had a high cohesiveness in the polymer matrix. This is attributed to the fact that these formulations have a higher glycerol content that acts as a plasticizer, giving it higher resistance to be dissolved [13,24]. In addition, these formulations contain a lower percentage of 90% starch, which gives them less capacity to retrograde due to the amylose content [18,19,25,26]. This would also be associated with the higher percentage of mucilage in the F2 and F4 formulations, as reported by Guadarrama-Lezama et al. [8], considering that mucilage has a highly branched structure with carboxyl groups, which gives it a higher ability to link and interact with the carboxyl and hydroxyl groups of starch [4,18].

Regarding the resistance to solubility, it was observed that the films subjected to a solution of strong acid (HCl, 0.01 M) and weak acid (CH_3_COOH, 0.01 M) showed similar behavior (Table 3), presenting between moderate and high solubility, the same as for the strong base (NaOH, 0.01 M). This fact is mainly due to the ease of establishing hydrophilic links of the hydrogen or Van der Walls type between the molecules of the solvent medium, hydrogen ion, and hydroxyls of the acid and base. The partial positive and negative charges that the functional groups of the film present, which are polar nature [8,27], especially glycerol, a small molecule, that is housed in the intermolecular matrix of the film, giving it permeable capacity [16,28,29]; therefore, when subjected to organic solvents, such as ethanol and ether, it was observed that the edible films did not show solubility due to their low or null polarity.

### 3.2. Water Activity

**Figure 1:**
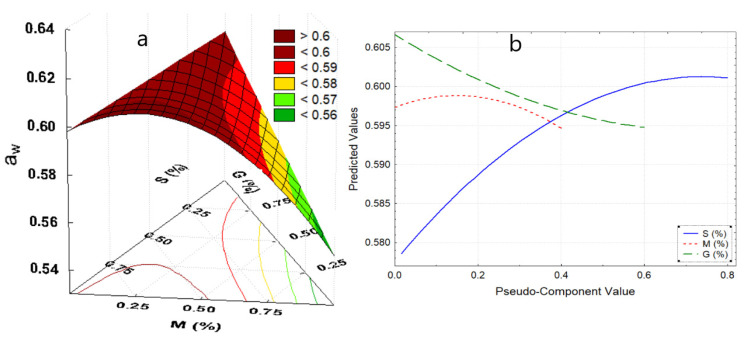
(**a**) Response surface for a_w_, (**b**) effects diagram for a_w_. Where: a_w_, water activity; S, starch; M, mucilage; G, glycerin.

Due to the components of the edible films, they would present a matrix with available carbonyl and hydroxyl groups, and probably sugar residues from potato starch and mucilage, increasing the available active hygroscopic sites which would increase the water activity (a_w_) [30,31]. In Table 4, a_w_ values ranging from 0.562 to 0.680 for edible film formulations are presented (*p*-value < 005). These values are adequate to inhibit the growth of microorganisms [6,32]. a_w_ was observed to increase slightly with the addition of potato starch and treatment temperature; because the hydrophilic polymeric chains of the starch swell, since they have more water linking groups coming mainly from amylopectin, starch would considerably influence water activity (Figure 1b), as mentioned by Ghanbarzadeh et al. [33], Kibar et al. [34], and Muscat et al. [35].

In contrast, the increase in mucilage decreases a_w_ (Figure 1b), this might be due to the fact that the galacturonic acid of the mucilage would not have undergone a substantial change in its structure during the synthesis of the film, since its linear structure makes it less receptive to water molecules [36,37]. This is while the addition of glycerol decreases a_w_ (Figure 1b), however Navia et al. [30], Tanada-Palmu et al. [38], Galus et al. [39], and Basiak et al. [40], reported a contrary behavior, this was attributed to the synergy of the components, such as nopal mucilage, and potato starch which is found in a higher percentage (Figure 1a).

### 3.3. FTIR Analysis

The FTIR spectra of potato starch and nopal mucilage are affected by the processes of mixing and heat treatment in the synthesis of edible films [41]. This has the capability to modify the molecular interactions of their constituents (stretching, bending, and twisting of chemical bonds), due to changes in the network of hydrogen bonds in the polymer matrix [20,42].

**Figure 2:**
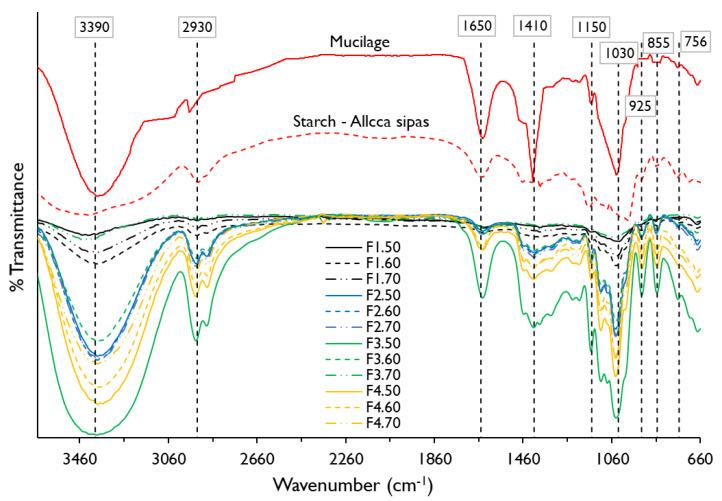
Fourier transform infrared (FTIR) spectra for potato starch, nopal mucilage, and edible films formulations.

In Figure 2, the spectra of starch of the native potato of the Allca Sipas variety and the nopal mucilage shows an intense band around 3390 cm^−1^ corresponding to an –OH stretching of the functional groups alcohol and carboxylic acids, and –NH of amides; on the other hand, there is a band around 2930 cm^−1^ that corresponds to an asymmetric stretch –CH_2_ being more pronounced for potato starch. Around the 1650 cm^−1^ band, the torsion and stretching –OH and C=O, which corresponds to bound water and amides, was observed as observed more intense for mucilage, and is due to the presence of proteins present [4,16]. The range between 1500 and 800 cm^−1^ is known as the fingerprint of material, and functional groups prevail –OH, C–O, –CH_2_, C–O–C, that includes molecules of sugars, alcohols, and organic acids [40,41] present in potato starch and nopal mucilage, being characteristic for these type of materials [8,10,18,40]. On the other hand, it was observed that the mucilage did not show the 925 cm^−1^ band corresponding to –OH deformation of carboxylic acids, as reported by Guadarrama-Lezama et al. [8].

When observing the spectrograms of the edible films, it is seen that the polymeric matrix of the different formulations maintain the predominant functional groups –OH, C–O, –NH, CH2, C=O, C–O–C from potato starch and nopal mucilage, however formulations F1.50, F1.60, and F1.70 do not show the 1410 and 1150 cm^−1^ band. This is justified by the low percentage of mucilage in these films (3%) and high starch content (Table 1), which is related to the behavior of water activity (Table 4, Figure 1b). To these bands, the potato starch shows slight stretching –OH, C–O, and C–O–C, so it could be assumed that starch has few ether and alcohol groups, as reported by Martinez et al. [18], which is influenced by the treatment temperature [7].

In the same way, it can be seen that all the films show –OH stretch in the 1030 cm^−1^ band (Table 5). This is due to the contribution of hydroxyl groups of the glycerol [43,44], which would promote dipole-dipole type hydrogen bonding interactions between starch, mucilage, and glycerol, proving their hydrophilic nature [9].

### 3.4. Thermal Analysis

**Figure 3:**
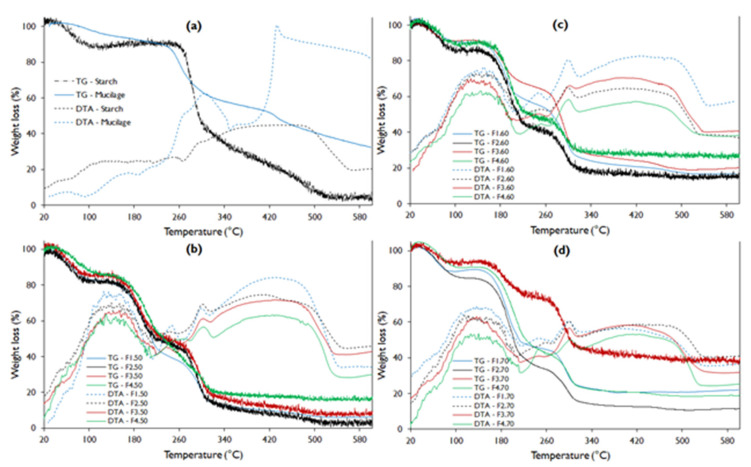
TGA-DTA curve for potato starch, nopal mucilage, and edible film formulations (**a**). Where: TG, thermogravimetric analysis curve; DTA, differential thermal analysis curve (**b**–**d**).

The TGA analysis shows that the native potato starch presented better thermal stability with a melting temperature (T_m_) close to 260 °C, while the mucilage showed T_m_ around 240 °C (Figure 3a), this difference is attributed to the fact that the starch has a higher number of free amino groups compared to mucilage (Figure 1). However, mucilage presents better stability to water loss, which manifests up to 80 °C, while starch barely up to 40 °C. This fact is due to the content of heteropolysaccharides (pectins) in the mucilage, so that this content makes it unstable at the fusion temperature [44,48,49].

While the water loss stability of edible films is very low, this is due to the high starch content in the formulations, as reported by Velásquez et al. [50] and López-García et al. [10]. However, F4 presents a slightly higher stability due to the high content of mucilage and glycerol.

The weight loss of the mucilage has three stages, the first comprising from 20 to 240.4 °C attributed to low molecular weight components, including water, through an endothermic process around 80 °C (gelatinization) observed in the curve DTA. A second stage occurs between 240.4 to 307.7 °C, with the exothermic process around 300 °C (Figure 3a), and a third that starts at 427.3 °C, with a tendency to continue decreasing above 600 °C (Table 6). This fact is possibly due to the decomposition of the mucilage polymeric matrix being resistant, due to its high molecular weight that oscillates between ranging from 13 × 10^6^ to 4.3 × 10^6^ Dalton [51], and the presence of minerals in the form of salts [10].

The potato starch presented a zone of 20 to 103.6 °C that is attributed to the loss of water representing 12.0% of weight, through an endothermic process around 70 °C (gelatinization), which is characteristic for these materials [19,21]. Another range from 103.6 to 299.2 °C in this zone, the decomposition of carbohydrates and peptides low molecular weight is considered, which represents 42.9% of weight. The third stage from 299.2 to 526.8 °C (41.8%) that corresponds to the material degradation zone, that is, the decomposition of the polymeric matrix of starch and high molecular weight polysaccharides which occurs above 320 °C [1,50,52].

Regarding the thermograms of the films, it is evident that they do not have a gelatinization zone in the DTA curve (Figure 3b–d) compared to starch and mucilage. This is because they present glycerin in their composition, which is a plasticizer. It is also observed that they present three well-defined sections corresponding to exothermic changes.

**Figure 4:**
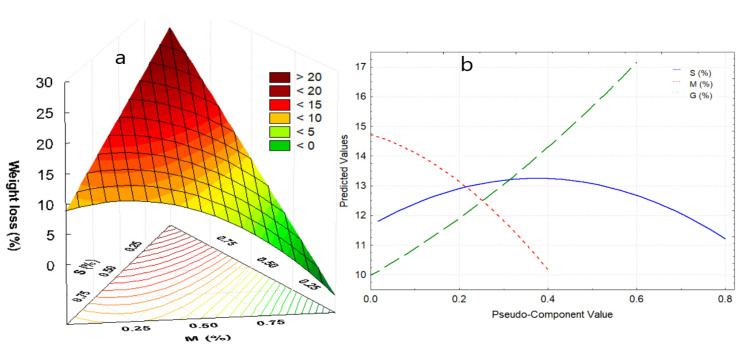
(**a**) Response surface for weight loss in the first stage, (**b**) effects diagram for weight loss in the first stage. Where: S, starch; M, mucilage; G, glycerin.

The first zone, between 20 to 116.9 °C, corresponds to the elimination of free and weakly bound water, which represents between 5.6 to 18.2% of the lost weight, which occurs around 100 °C for films. The weight loss is mainly due to the addition of glycerin, and to a medium extent to the increase in starch (Figure 4a). This fact is attributed to a large number of active sites with –OH groups that they possess, conferring high hygroscopic capacity. On the other hand, the addition of nopal mucilage allows the maintenance of humidity in the edible film (Figure 4b).

In the second stage, the decomposition of carbohydrates and peptides occurs in a second exothermic stage between 116.9 and 236.8 °C, where the films maintain their structure and the C–O, –OH, –COO– interaction bonds are found still constituted. At this stage, an intermediate plateau that corresponds to changes in characteristic polymorphism in biopolymers is observed in all TGA curves [1,53,54], and is due to the existence of different conformers of the same molecule. A third exothermic stage ranging from 236.8 to 321.1 °C, in which the decomposition of polysaccharides (pectins, cellulose, fibers) and high molecular weight proteins occurs, that is, the decomposition of functional groups of tertiary amines and carbonyls (as evidenced in the spectrograms of Figure 1), giving rise to the formation of CO_2_, NO_2_, and SO_2_, with oxidation and total decomposition of organic matter [55,56].

Above 500 °C, a stabilization of the DTA curve is evident, which corresponds to endothermic crystallization that allows the formation of mineral salts and ashes [50,57]. This represents 13.9 to 23.8% of the residues, and a similar behavior was reported by Dick et al. [1], López-García et al. [10], and Guadarrama-Lezama et al. [8].

Thermograms show that F2 formulations reported higher water loss (Figure 3b–d), and that it decreases with treatment temperatures from 18.2 to 14.7% (Table 6), while formulation F3 reported lower weight loss values from 13.8 to 5.6%. This fact is attributed to the glycerin content, presenting an inverse effect with the weight loss of the film, as observed by López-García et al. [10]. 

When the starch proportion increases, there is a great loss of weight in the first stage, and the same behavior was observed for the second stage. In the third stage, polysaccharides and molecules of high molecular weight are decomposed, which is attributed to starch and part of mucilage, and correspond to formulation F1 (94% starch and 3% glycerol) and F3 (93% starch and 2 % of glycerol); while the maximum amount of weight loss was obtained for formulation F1 and the lowest for F4 (90% starch, 5% mucilage, 5% glycerol). That is to say, the higher the content of mucilage in the film, the less weight loss occurs, since it could present more mineral salts. The same behavior was observed by Guadarrama-Lezama et al. [8], López-García et al. [10], and Gheribi et al. [58].

### 3.5. SEM Analysis

**Figure 5:**
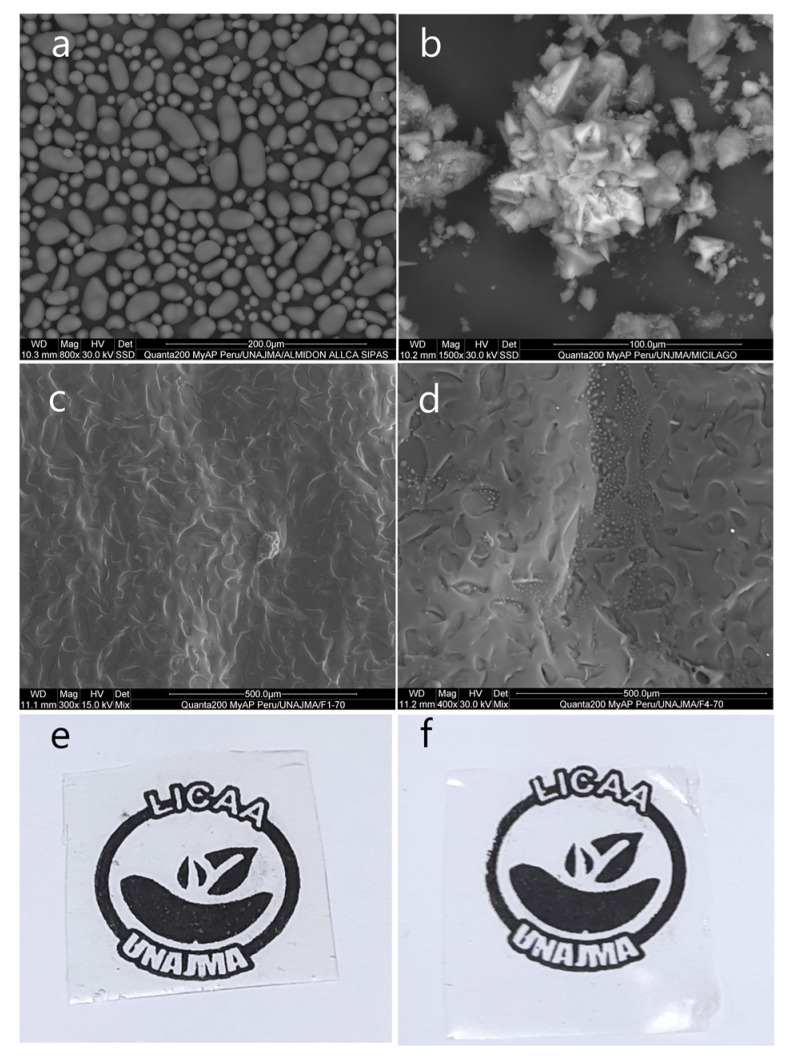
(**a**) SEM image for native potato starch Allcca sipas, (**b**) SEM image for dehydrated nopal mucilage, (**c**) SEM image for F1-70, (**d**) SEM image for F4-70, (**e**) edible film F1-70, (**f**) edible film F4-70.

In Figure 5, the photomicrograph of the native potato starch Allcca sipas is observed, whose granules have oval shapes of different sizes less than 25 µm, while the dehydrated nopal mucilage presented in the form of amorphous crystals. This is due to the content of calcium and potassium salts [59].

SEM analysis was considered for the films whose formulations were extreme, F1-70 (94% S, 3% M, 3% G) and F4-70 (90% S, 5% M, 5% G), and it was observed that the films were compact, translucent, and without cracks in both formulations (Figure 5e,f). However, the surfaces presented with indentations which were smaller for F1-70 (Figure 4), and this is due to the loss of water during the thermomolding process, and related to the higher starch content, which traps more water, as reported by Zhao et al. [20], Andreuccetti et al. [22], Tosif et al. [53], Andreuccetti et al. [60], and Soukoulis et al. [61]. On the other hand, small bubbles were observed in F4-70, and this is because the mucilage retains interstitial water in the film. Furthermore, during the thermomolding process, the water tries to escape by steam action, however the plasticizing effect of glycerin and starch act as a barrier in the film matrix, forming small pockets [62,63,64].

## 4. Conclusions

The increase in mucilage of nopal and glycerol in edible films decreases the solubility, while the addition of potato starch and the increase in the synthesis temperature greatly increases the solubility, being between medium and high solubility in acidic medium, basic and water, and completely insoluble in non-polar solvents. Edible films show adequate values of water activity for packing food, which increases significantly with the addition of potato starch and treatment temperature, while the addition of mucilage and glycerol slightly decrease a_w_. The FTIR analysis showed the interaction of the components of the edible films, preserving their functional groups and being considerably influenced by the addition of nopal mucilage. The TGA-DTA study revealed that the edible films have low thermal stability, retain water around 100 °C, and that the higher starch content reported greater weight loss by thermal decomposition, with the opposite happening with the addition of mucilage. The found results allow us to establish that the synthesized edible films can be used as a packing material, besides having an attractive nutritional contribution due to the conservation of their constituents and functional groups, and have adequate thermal stability as a food coating.

## Figures and Tables

**Table 1:** Formulation of edible films.

Formulation	S (%)	M (%)	G (%)	T (°C)
F1.50	94	3	3	50
F1.60	94	3	3	60
F1.70	94	3	3	70
F2.50	91	4	5	50
F2.60	91	4	5	60
F2.70	91	4	5	70
F3.50	93	5	2	50
F3.60	93	5	2	60
F3.70	93	5	2	70
F4.50	90	5	5	50
F4.60	90	5	5	60
F4.70	90	5	5	70

Where: F, formulation; S, starch; M, mucilage; G, glycerin; T, temperature.

**Table 2:** Solubility of prepared edible films in different media (%).

	Basic Medium (pH = 8.7)				Acidic Medium (pH = 4.7)				Ultrapure Water (pH = 7.0)			
	$\bar{x}$	±	s	*	$\bar{x}$	±	s	*	$\bar{x}$	±	s	*
F1.50	35.92	±	1.03	d	34.46	±	0.74	d	34.96	±	0.50	d
F1.60	40.36	±	0.47	e	40.69	±	0.89	d,e	38.15	±	0.96	e
F1.70	40.42	±	0.62	a,b	39.17	±	1.03	b	38.51	±	0.18	b
F2.50	26.87	±	1.72	c	23.01	±	1.15	c	19.77	±	2.03	c,d
F2.60	29.30	±	0.56	e	31.29	±	0.78	e,f	27.75	±	0.65	e
F2.70	29.67	±	0.34	f	28.85	±	0.38	g	28.54	±	1.51	f
F3.50	47.87	±	1.60	c	48.43	±	2.37	c	50.19	±	1.93	c
F3.60	51.65	±	1.45	b	54.08	±	3.69	b	53.03	±	0.54	a,b
F3.70	50.71	±	0.77	e	48.63	±	1.29	d,e	48.02	±	1.27	e
F4.50	24.03	±	1.68	e,f	21.96	±	0.67	g	22.41	±	0.72	f
F4.60	28.34	±	1.20	a	30.31	±	1.33	a	26.31	±	0.66	a
F4.70	28.57	±	1.54	e	24.72	±	1.57	f,g	27.46	±	1.49	e

Where: F, formulation; $\bar{x}$, mean; s, standard deviation. * Different letters indicate significant difference, evaluated through the Tukey test at 5% significance.

**Table 3:** Solubility resistance in edible films.

	HCl * (0.01 M)	CH_3_COOH ** (0.01 M)	NaOH *** (0.01M)	Ethanol	Ether	Ultrapure Water
F1.50	MS	MS	MS	NS	NS	MS
F1.60	HS	HS	MS	NS	NS	MS
F1.70	HS	HS	MS	NS	NS	MS
F2.50	MS	MS	MS	NS	NS	LS
F2.60	MS	MS	MS	NS	NS	MS
F2.70	MS	MS	MS	NS	NS	MS
F3.50	HS	HS	HS	NS	NS	HS
F3.60	HS	HS	HS	NS	NS	HS
F3.70	HS	HS	HS	NS	NS	HS
F4.50	MS	MS	MS	NS	NS	MS
F4.60	MS	MS	MS	NS	NS	MS
F4.70	MS	MS	MS	NS	NS	MS

Where: HS, high solubility; MS, moderately soluble; LS, low solubility; NS, non soluble. * Hydrochloric acid, ** acetic acid, *** sodium hydroxide.

**Table 4:** Water activity of prepared edible films.

Formulation	a_w_			*
F1.50	0.578	±	0.007	d
F1.60	0.619	±	0.002	b
F1.70	0.596	±	0.005	c
F2.50	0.613	±	0.005	b
F2.60	0.576	±	0.004	d
F2.70	0.586	±	0.003	c,d
F3.50	0.562	±	0.006	e
F3.60	0.639	±	0.003	a
F3.70	0.615	±	0.002	b
F4.50	0.580	±	0.007	d
F4.60	0.573	±	0.002	d,e
F4.70	0.580	±	0.002	d

Where: F, formulation; a_w_, water activity. * Different letters indicate significant difference, evaluated through the Tukey test at 5% significance.

**Table 5:** FTIR spectra for formulations of edible films, starch, and mucilage.

Wavenumber (cm^−1^)	Functional Group	Vibration Type	Compound Type	Present in
3390	–NH y –OH	Stretching	Alcohols, secondary amide, and carboxylic acids	All formulations, starch, and mucilage
2930	–CH_2_–	Asymmetric stretching	Methylene groups	All formulations, starch, and mucilage
1650	–OH, C=O	Bending, stretching	Water, amide	F2.50, F2.60, F2.70, F3.50, F3.60, F3.70, F4.50, F4.60, F4.70, starch, and mucilage
1410	–OH, C–O	Stretching	Alcohols	F2.50, F2.60, F2.70, F3.50, F3.60, F3.70, F4.50, F4.60, F4.70, starch and mucilage
1150	C–O–C	Stretching	Ether	All formulations, starch, and mucilage
1030	C–O	Stretching	Alcohols	All formulations, starch, and mucilage
925	–OH out of plane	Deformation	Carboxylic acids	All formulations, and starch
855	–CH2–	Deformation	Methylene groups	All formulations, starch, and mucilage
756	C–O–C	Stretching	Ether	All formulations, starch, and mucilage

Source: Hu et al. [45], Galicia-García et al. [46], Arief et al. [47].

**Table 6:** Weight loss and decomposition temperature, determined by TGA—DTA.

	First Stage		Second Stage		Third Stage		Residue (%)	Max. Weight Loss (%)
	Weight Loss (%)	T (°C)	Weight Loss (%)	T (°C)	Weight Loss (%)	T (°C)		
Starch	12.0	103.6	42.9	299.2	41.8	526.8	3.2	96.8
Mucilage	11.2	240.4	25.3	307.7	12.8	427.3	50.7	49.3
F1.50	16.1	91.4	41.3	226.9	27.9	313.5	14.8	85.2
F1.60	8.6	92.4	29.8	206.1	34.4	312.1	27.2	72.8
F1.70	11.1	80.8	40.0	215.4	26.2	313.9	22.7	77.3
F2.50	18.2	90.8	31.4	218.2	36.5	316.2	13.9	86.1
F2.60	15.2	102.8	41.6	229.6	24.6	321.1	18.7	81.3
F2.70	14.7	103.6	43.0	212.8	26.4	314.1	15.9	84.1
F3.50	13.8	92.9	36.5	236.8	28.8	319.5	20.9	79.1
F3.60	8.2	85.2	22.2	213.2	37.5	310.9	31.2	68.8
F3.70	5.6	81.9	18.1	219.5	29.9	317.6	46.4	53.6
F4.50	14.1	116.9	31.2	216.9	34.1	314.3	20.6	79.4
F4.60	11.8	108.3	36.5	216.1	22.4	316.4	29.3	70.7
F4.70	9.3	104.4	41.9	227.4	25.1	318.0	23.8	76.2

Where: F, formulation; T, temperature.

## Data Availability

The data presented in this study are available in this same article.
